# Fluorescence Imaging of Mitochondrial Redox State to Assess Diabetic Wounds

**DOI:** 10.1109/JTEHM.2019.2945323

**Published:** 2019-10-18

**Authors:** Shima Mehrvar, Kevin T. Rymut, Farnaz H. Foomani, Soudeh Mostaghimi, Janis T. Eells, Mahsa Ranji, Sandeep Gopalakrishnan

**Affiliations:** 1 Biophotonics LabDepartment of Electrical EngineeringUniversity of Wisconsin Milwaukee 14751 Milwaukee WI 53211 USA; 2 College of NursingUniversity of Wisconsin Milwaukee 14751 Milwaukee WI 53211 USA; 3 Department of Biomedical SciencesUniversity of Wisconsin Milwaukee 14751 Milwaukee WI 53211 USA

**Keywords:** Diabetes, mitochondria, optical imaging, redox state, wound healing

## Abstract

*Background:* Diabetes is known to cause delayed wound healing, and chronic non-healing lower extremity ulcers may end with lower limb amputations and mortalities. Given the increasing prevalence of diabetes mellitus worldwide, it is critical to focus on underlying mechanisms of these debilitating wounds to find novel therapeutic strategies and thereby improve patient outcome. *Methods:* This study aims to design a label-free optical fluorescence imager that captures metabolic indices (NADH and FAD autofluorescence) and monitors the *in vivo* wound healing progress noninvasively. Furthermore, 3D optical cryo-imaging of the mitochondrial redox state was utilized to assess the volumetric redox state of the wound tissue. *Results:* The results from our *in vivo* fluorescence imager and the 3D cryo-imager quantify the differences between the redox state of wounds on diabetic mice in comparison with the control mice. These metabolic changes are associated with mitochondrial dysfunction and higher oxidative stress in diabetic wounds. A significant correlation was observed between the redox state and the area of the wounds. *Conclusion:* The results suggest that our developed novel optical imaging system can successfully be used as an optical indicator of the complex wound healing process noninvasively.

## Graphical abstract

This paper designed a label-free optical fluorescence imager that captures metabolic indices (NADH and FAD autofluorescence) and monitors the in vivo wound healing progress noninvasively. The results quantify the differences between the redox state of wounds on diabetic mice in comparison with the control mice.

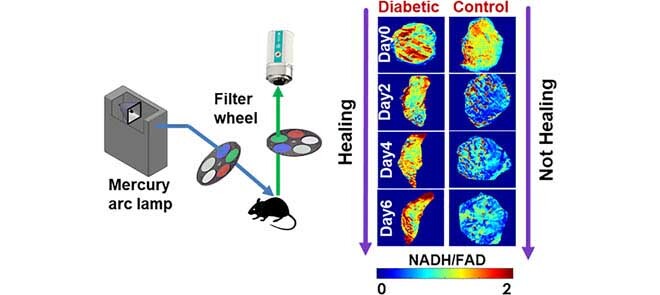

## I. Introduction

Chronic lower-extremity ulcers are a common complication of diabetes, and approximately 15% to 25% of individuals diagnosed with diabetes will develop a chronic wound at some point in their lifetime [1]. Diabetic ulcers can be a leading cause of disability and mortality when wound healing does not progress normally, causing a significant burden on the health-related quality of life (HRQoL) for patients and their caregiver, which contributes major costs to healthcare systems and societies [2]–[5]. Currently, symptomatic evaluation, wound area monitoring, and swab-based assays are the only clinical approaches to diagnose and monitor chronic wounds [6], [7]. Due to the dysregulated healing process, the current strategies of diabetic wound care have a limited efficacy [4]. Therefore, there is a critical need to elucidate mechanisms of physiologic wound healing and to develop new biomarkers and diagnostic tools to evaluate and quantify wound status that can guide the clinical care [8], [9].

Normal wound healing is a critical biological function required to maintain the barrier function of skin. The process of normal wound healing involves complex and dynamic overlapping phases of hemostasis, inflammation, tissue proliferation, and tissue remodeling [10], [11]. However, in diabetic wounds, each of these phases is compromised, disorganized, and uncoordinated, delaying the orderly progression of healing [12]. The mechanisms of the underlying pathogenesis of impaired diabetic wound healing are still unclear. The profound delay in diabetic wound healing could be a net result of micro- and macrovascular disease [13], neuropathy and sensory loss, inadequate angiogenesis [14], and a diminish in fibroblast activities and epidermal barrier function [9].

Increased oxidative stress has been shown to be a major contributor to diabetic complications, including retinopathy [15], [16], nephropathy [17], and cardiovascular disease [18]. Oxidative stress also plays a significant role in regulating wound healing [19], and the resulting redox imbalance has major implications in diabetic wounds [11]. Hyperglycemia disrupts mitochondrial electron transport, resulting in a profound increase in reactive oxygen species and intracellular oxidative stress [20], [21]. The energy required for ATP synthesis in mitochondria is derived from the oxidation of two key coenzymes, nicotinamide adenine dinucleotide (NADH) and flavin adenine dinucleotide (FADH2). NADH and FADH are produced during glycolysis and the citric acid cycle. Mitochondrial complexes can be assessed with commercially available biochemical assays using isolated mitochondria. However, NADH and FAD are autofluorescent and can be captured by optical imaging techniques in a more “native” condition [22].

The development and utilization of optical imaging technologies is an exciting field in dermatology to report tissue structure, activity, and physiology [23]. Optical techniques such as fluorescence spectroscopy [24], [25], thermal imaging [26], perfusion imaging [27], optical coherence tomography [28] and multiphoton microscopy [29] have been utilized to assess various biomarkers of wound healing in skin. Fluorescence metabolic imaging techniques pioneered by Chance et al. [30] have been developed to measure mitochondrial bioenergetics (NADH and FAD). Fluorescence spectroscopy of metabolic indices measures optical biopsies from the surface of tissues *in vivo* and *ex vivo* [31]–[33]. Fluorescence cryo-imaging is another optical metabolic imaging technique, which provides a 3D metabolic state of the tissue. Assessment at cryogenic temperature provides higher quantum yields [32], [34], [35].

In this study, we employed optical imaging systems to quantitatively assess metabolic activity and oxidative stress within cells and tissues, enabling us to determine whether altered mitochondrial metabolism, redox state, is a key contributor to nonhealing diabetic wounds. The ratio of these fluorophores, (NADH/FAD), called redox ratio, acts as a quantitative marker of the mitochondrial redox state of the tissue [32], [34]. We developed and introduced a custom-designed *in vivo* fluorescence imager system with the capacity to assess wound healing progress and quantify the mitochondrial redox state (NADH/FAD) of the wound noninvasively in a diabetic mice model. This unique and novel device evaluates real-time metabolic images (NADH and FAD) of the wounds. Furthermore, we utilized a 3D optical cryo-imaging system to obtain redox information from the wound tissue and see whether it correlates with the real-time surface information captured by the *in vivo* fluorescence imager system. To further strengthen the imaging studies, we also evaluated the wound healing process on diabetic mice by histological staining.

## II. Materials & Methods

### A. Experimental Protocol

#### 1) Animals

Studies were conducted in genetically diabetic mice and non-diabetic controls. Animal use protocols were approved by Institutional Animal Care and Use Committee (IACUC, Protocol #16–17#12) at the University of Wisconsin Milwaukee, and experiments were conducted in accordance with the National Institute of Health, Guide for the Care and Use of Laboratory Animals. Genetically diabetic male and female ~20-week old mice (db/db; BKS.Cg-m+/+ Leprdb) were obtained from Jackson Laboratories (Bar Harbor, ME) and were housed under conditions of controlled temperature and illumination in an animal care facility with a 12-hour light/dark cycle throughout the acclimation (4 weeks) and test periods. Efforts made to minimize animal suffering and to reduce the number of animals in experiments. Age-matched non-diabetic B6/J mice were used as the controls. We used half male and female in each group to study wound healing in both sexes, and a total of 12 mice per group were sacrificed in this study. Blood glucose concentrations and the bodyweight of the mice were measured weekly. The diabetic mice weigh 52.5 gr on average, and blood glucose concentrations ranged from 423–600 mg/dL, which is much higher than the blood glucose level in B6/J mice.

#### 2) Wound Incision Model

Mice were anesthetized with isoflurane (4% isoflurane in 100% oxygen at a flow rate of 1 L/min). The dorsal area was shaved, and a 10mm circular full-thickness wound was prepared midline at the shoulder-level. Wounds were created by cutting through the skin and panniculus carnosus with surgical scissors. The analgesic, carprofen (5 mg/kg, sc), was administered to all animals before making the incision. After the surgery, animals were individually caged and placed on Delta phase isothermal pads (Braintee Scientific, Inc) until fully recovered. Carprofen (5 mg/kg, sc) was administered at 24- and 48-hours post-surgery to ameliorate the discomfort, and mice were monitored twice daily for manifestations of pain.

#### 3) Timeline and Tissue Processing

Table 1 shows the experimental protocol timeline. The *in vivo* images of metabolic indices (NADH and FAD) were captured immediately following the wound incision. For longitudinal assessment of wound healing, the imaging was also performed on the $2^{\mathrm {nd}}$, $4^{\mathrm {th}}$, and $6^{\mathrm {th}}$ day of post-wounding (n = 6/group). At the end of *in vivo* experiments (day 6), the mice were euthanized by CO2 inhalation, and the entire wound biopsies were collected and snap-frozen in liquid nitrogen for generating day 6 cryo-imaging data. Wound biopsies from 4 mice per group at day 0 of wound induction were harvested. The cryo-images were acquired only at day 0 and day 6 because the maximum difference in 3D cryo-images are from the first and last point, and we had to sacrifice the animal for complete sample excision. For histology assessment, 2 diabetic mice were euthanized at day 0 and day 6 of post wounding, and the entire wound, including 2 mm of the wound edge, was excised *en bloc*, frozen in OCT at −80°C, and processed for histology as described by [36].

**TABLE 1:** Experimental Protocol Timeline

Activity^a^	Days after wound induction			
	Day0	Day2	Day4	Day6
$In ~vivo$ fluorescence imaging				
Cryo-imaging^b^				
Histology^b^				

^a^ Each activity was conducted only at the colored days.

^b^ For wound biopsies, the animals were euthanized.

### B. $In ~vivo$ Fluorescence Imager

#### 1) Instrumentation

**FIGURE 1.**
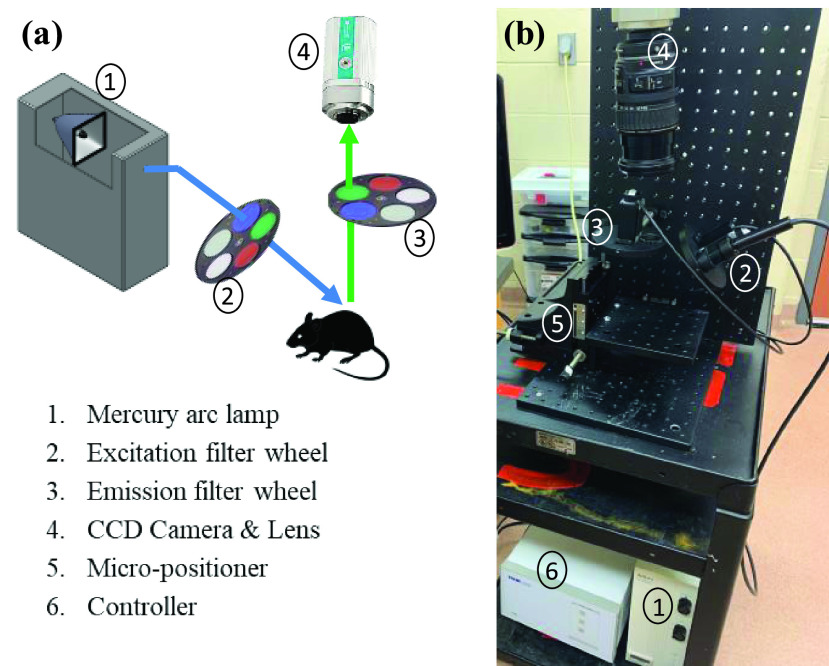
(a) Schematic view of in vivo fluorescence imager. (b) physical implementation of the system in the lab set-up.

Fig. 1 illustrates a schematic representation and the physical implementation of the designed surface fluorescence imager. This system can record the online and real-time fluorescence images of tissues using a charge-coupled camera (QImaging, Rolera EM-C^2^, 14 bit) with 1,004 $\times$ 1,002 pixel arrays. A mercury arc lamp (Intensilight, Nikon, Tokyo, Japan) generates the excitation light through a liquid light guide. For each channel, the light spectrum is filtered by optical filters at selected wavelengths to excite the specific fluorophores from the surface of the wounds. For mitochondrial redox experiments, we set the excitation filter for NADH at 350 nm (80-nm bandwidth, UV Pass Blacklite, HD Dichroic, Los Angeles, CA). The FAD excitation filter was set at 437 nm (20-nm bandwidth, 440QV21, Omega Optical, Brattleboro, VT). NADH and FAD emission filters are set at 460 nm (50-nm bandwidth, D460/50M, Chroma, Bellows Falls, VT) and 537 nm (50-nm bandwidth, QMAX EM 510–560, Omega Optical), respectively. Two neutral density filters (ThorLabs, NJ) are used as excitation and emission filters for white light channel imaging. The appropriate excitation and emission filters are selected using two motorized filter wheels (FW103H, ThorLabs, NJ), which is controlled by a two-channel APT™benchtop stepper motor controller (BSC202, ThorLabs, NJ). An XYZ translational micro-positioner (ThorLabs, NJ) is used to control the stage movement and focus of the images.

#### 2) Image Processing

**FIGURE 2.**
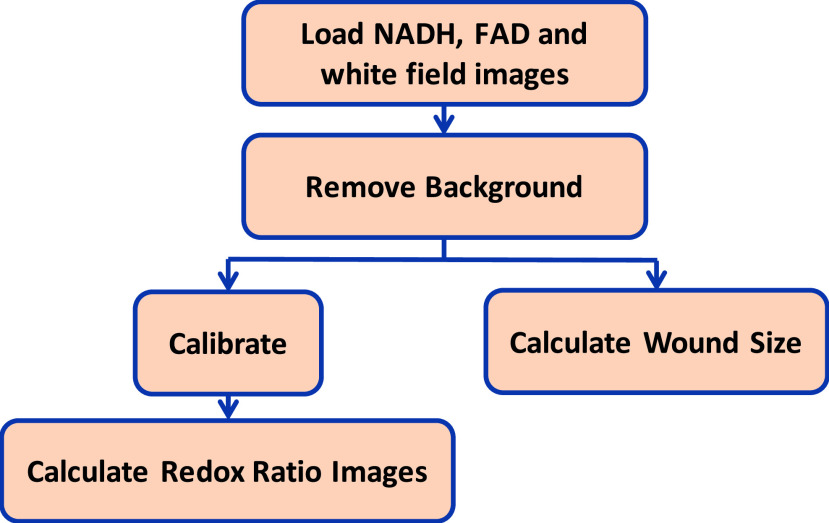
Flowchart of the algorithm for processing the *in vivo* fluorescent images.

NADH and FAD autofluorescence images of wounds were analyzed using MATLAB (Fig. 2). To segment the wound from the background skin tissue, we used white light images and found the wound area visually. The binary mask that is created from the white light image has been used to segment the wound region in NADH and FAD images by setting the background to zero. The intensities of the NADH and FAD images were calibrated to minimize day-to-day variations in light intensity. For calibration, we captured images from a cuvette containing $50\mu \text{M}$ NADH solution in NADH channel of imaging and $0.5\mu \text{M}$ FAD solution in FAD channel of imaging. Then, the wound NADH and FAD images were normalized to the average intensity of cuvette images. The ratio of the autofluorescent images (NADH/FAD) was calculated pixel-by-pixel. Subsequently, the mean of redox ratio histograms was considered as the quantitative marker and calculated according to (1),

\begin{equation*} SurfaceRR=\frac {1}{N}\sum \nolimits
                _{1}^{N} {(wound\,pixels(n))}\tag{1}\end{equation*}

where N is the number of wound pixels. For quantifying the wound closure, the wound area can be calculated from the same metabolic images and can be approximated as the number of pixels within the wound multiplied by the pixel size. Therefore, the normalized wound area at day t is calculated as follows,

\begin{equation*} {Normalized} {Wound} {Area} =\frac
                {PS(t)\times N(t)}{PS(0)\times N(0)}=\frac {N(t)}{N(0)}\tag{2}\end{equation*}

where N(t) is the number of wound pixels at day t. N(0) is the number of wound pixels at day 0, i.e., the initial wound size. PS(t) is the pixel size at day t and equals to $40\mu \text{m}$, a constant number. Therefore, we can simplify the normalized wound size as the ratio of the number of pixels at day t divided by the number of pixels at day 0.

### C. 3D Fluorescent Cryo-Imager

The 3D fluorescence cryo-imager system was custom-designed in Biophotonics Laboratory at the University of Wisconsin Milwaukee. This system preserves the metabolic state of the tissue by maintaining the flash-frozen sample at −80°C enabling us to visualize a snapshot of the mitochondrial redox state at the time of freezing. A complete description of the system can be found in our previous cryo-imaging studies [32], [35]. Briefly, a mercury arc lamp (200 W lamp, Oriel, Irvine, CA, in the light source from Ushio Inc., Japan) is used as the light source. Appropriate optical filters at selected wavelength are utilized to excite the specific fluorophores from the surface of the frozen tissue. For NADH channel, excitation filter and emission filters are set at 350 nm (80-nm bandwidth, UV Pass Blacklite, HD Dichroic, Los Angeles, CA) and 460 nm (50-nm bandwidth, D460/50M, Chroma, Bellows Falls, VT), respectively. The excitation and emission filters for FAD channel are set at 437 nm (20-nm bandwidth, 440QV21, Omega Optical, Brattleboro, VT) and 537 nm (50-nm bandwidth, QMAX EM 510–560, Omega Optical), respectively. All filters are controlled by two motorized filter wheels (Oriental Motor Vexta Step Motor PK268-01B). The emitted autofluorescent signals are captured with the image recordings system (CCD camera, QImaging, Rolera EM-C2, 14 bit). The NADH and FAD fluorophores excited and detected sequentially and separately. Their emission spectra do not overlap with each other, which allows for selective detection of fluorescence between the two fluorophores.

Similar to the *in vivo* fluorescence imager, the images obtained from 3D cryo-imager were analyzed using a code written in MATLAB. Calibration was performed using a flat field image at both NADH and FAD channels. The background low-intensity voxels were set to zero, and 3D rendered redox ratio image (NADH/FAD) were calculated voxel-by-voxel. Equation (3) finds volumetric RR by calculating the volumetric mean value of redox ratio histograms,

\begin{equation*} VolumetricRR=\frac {1}{N_{v}}\sum \nolimits
              _{1}^{n}\, {(wound~voxels(n))}\tag{3}\end{equation*}

where $N_{v}$ is the number of wound biopsy voxels.

### D. Statistical Analysis

The Surface RR, Volumetric RR, and Normalized wound area in two groups of mice (Diabetic and Control) were assessed using two-factor repeated ANOVAs. Tukey’s post hoc analysis was used with the significant criteria p-value<0.05. For correlations between measurements, a Pearson correlation coefficient was determined. Statistical analysis was performed using MATLAB.

### E. Histological Evaluation of Wound Tissue

The histological evaluation of the wound tissues was performed on hematoxylin and eosin (H&E)-stained sections, according to previously published [36].

## III. Results

### A. $In ~vivo$ Fluorescence Imaging of Wound Healing

**FIGURE 3.**
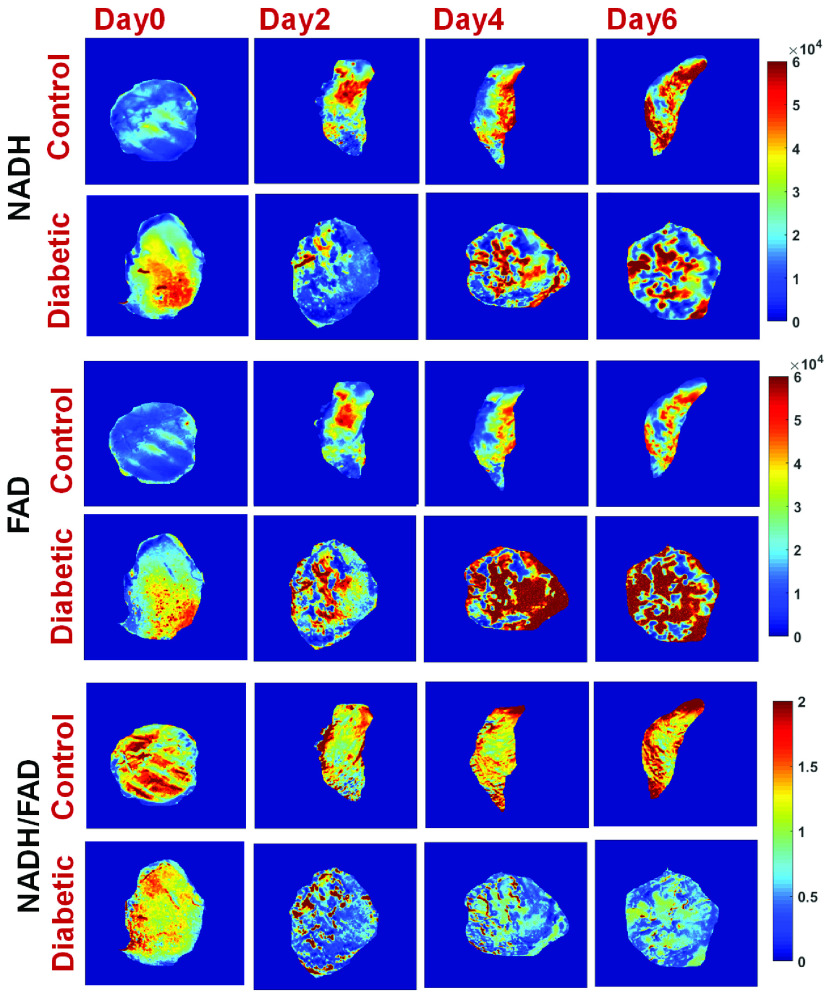
*In vivo* fluorescence images of NADH and FAD, and the tissue redox ratio (NADH/FAD) for representative wounds are shown over days. Unlike the wound on the control mice, the diabetic wounds showed a decrease in the NADH intensity and an increase in the FAD intensity, therefore a decrease in RR (bottom row).

**FIGURE 4.**
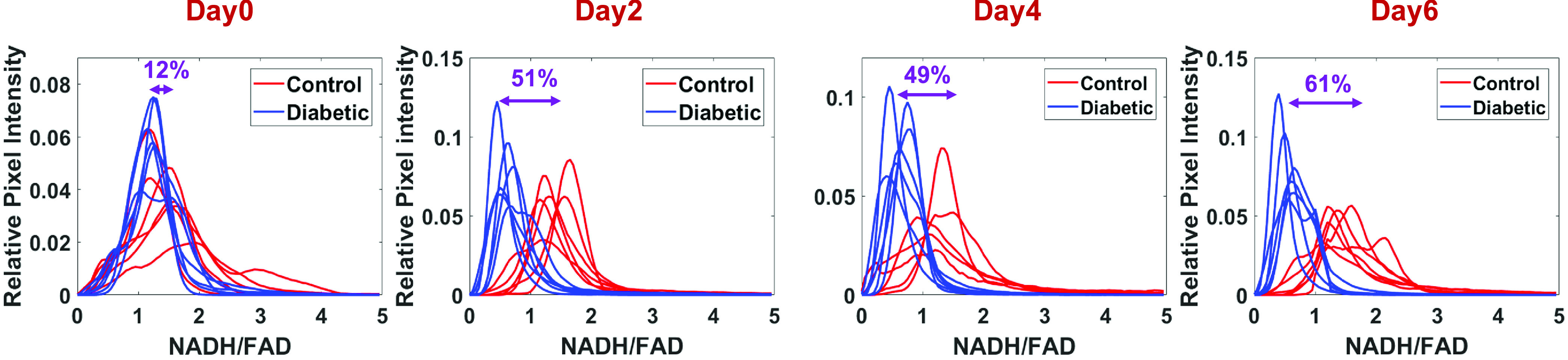
Intensity histogram distribution of redox ratio (NADH/FAD) for the diabetic vs. nondiabetic wounds over time. The percentage difference between the mean value of histograms (Surface RR) from control vs diabetic is shown for each day of post-punching (n = 6/group).

The *in vivo* fluorescence imager was used to evaluate temporal changes in the fluorescence images (NADH, and FAD). The redox ratio (NADH/FAD) of excisional wounds on diabetic and non-diabetic mice was calculated over 6 days of post-wounding (Fig. 3). Their corresponding histograms are shown in Fig. 4, demonstrating the differences in the redox state of the two groups longitudinally. Comparing diabetic mouse to control, lower NADH and higher FAD fluorescence signals were observed within the diabetic wound, resulting in a 61% drop in redox ratio value (oxidized state) at day 6 relative to day 0. The histograms of the surface redox ratio show that the diabetic wounds had a lower redox ratio compared to controls starting at day 2.

### B. 3D Cryo-Imaging of Volumetric Redox State

**FIGURE 5.**
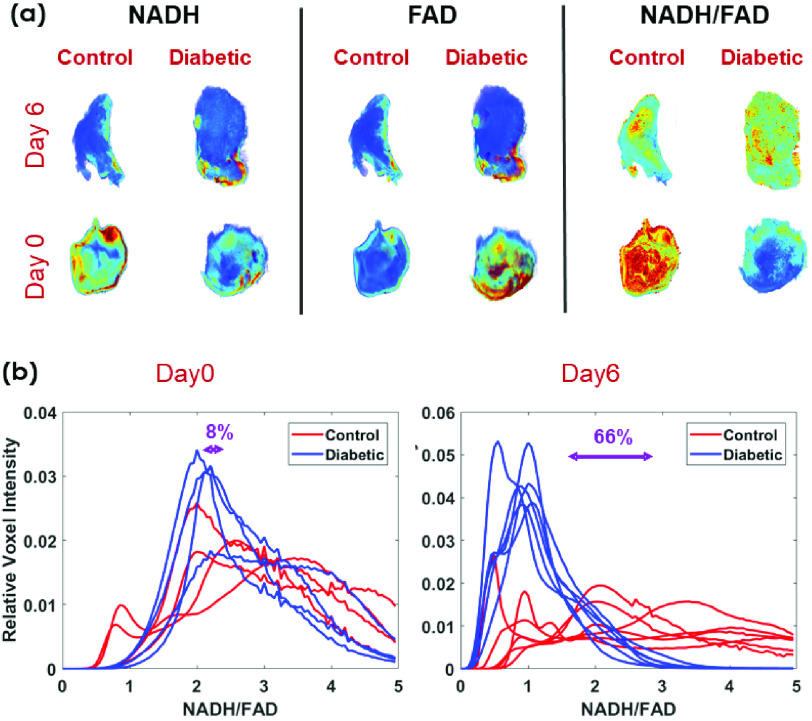
(a) 3D cryo-images of representative biopsy wounds from diabetic vs. controls at day0 and day6 of post punching, (b) their corresponding tissue redox ratio (NADH/ FAD) histograms. The percentage difference between the mean value of histograms (Volumetric RR) from control vs diabetic is shown for each day of post punching. (n = 4/group for Day 0 mice and n = 6/group for Day 6 mice). The volumetric RR of diabetic wounds dropped by 66% at day 6 in comparison with the wounds on the control mice (p-value< 0.001).

Fig. 5a shows 3D rendered metabolic images (NADH, FAD, and redox ratio) obtained from representative diabetic and control wounds at the beginning and the end of the experimental protocol. Fig. 5b shows the histogram comparison of the volumetric redox ratio. Complying to the trend that we observed with the *in vivo* fluorescence imager, there were no significant differences between the two groups of wound biopsies at the induction day. However, at the end of the experimental protocol, the diabetic mice had %66 lower volumetric redox ratio (oxidized state) comparing to controls, i.e., lower NADH and higher FAD fluorescence signals.

### C. Statistical Analysis

**FIGURE 6.**
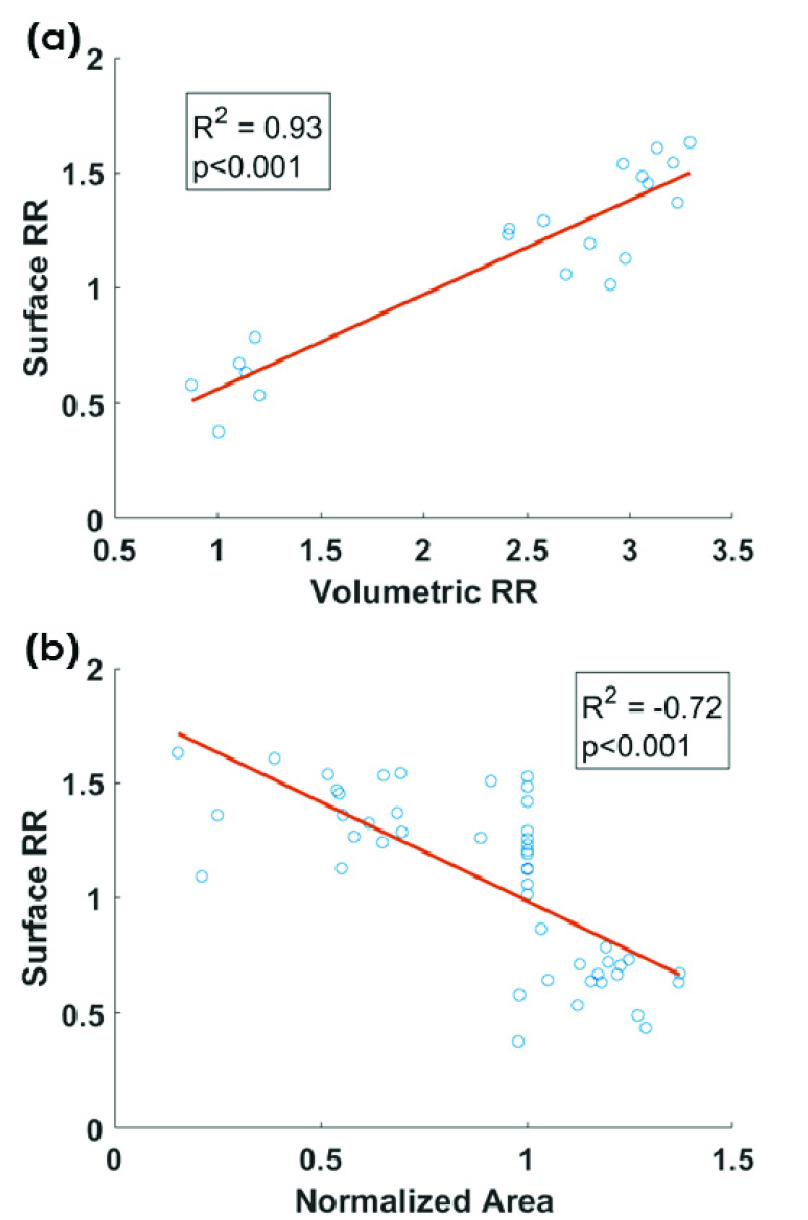
(a) Correlation between metabolic markers: Surface RR (measured by in vivo fluorescence imager) and volumetric RR (measured by cryo-imager). Circles are the data at day 0 and day 6 post wounding, and the straight line is the linear regression of the data. (b) correlation between surface RR and area of the wounds. Circles are *in vivo* data during experimental protocol.

The surface RR were calculated using (1), and their means ± standard error (SE) are shown in Table 2 to display the temporal redox state trend of wounds (n = 6/group). The normalized wound area, a quantification marker for the longitudinal wound closure, showed a drop for normal wound healing in non-diabetic mice while the diabetic wounds showed no sign of wound closure over the 6 days following wound application. The temporal difference between diabetic and non-diabetic wounds was not significant at the day of wound induction. However, at day 2, 4, and 6 post-wounding, the redox state of wounds was significantly different (p-value<0.001). Similarly, the mean ± SE of volumetric RR shows a significant difference between the two groups at day 6 of post-wounding (Table 2). The surface RR has a significant correlation with volumetric RR (Fig. 6a). Also, there was a significant negative correlation between the surface RR and the normalized wound area (Fig. 6b).

**TABLE 2:** Longitudinal Wound Assessments

		DAY0	DAY2	DAY4	DAY6
SURFACE RR^A^	Control	1.32 ± 0.08	1.37 ± 0.05	1.27 ± 0.06	1.52 ± 0.04
	Diabetic	1.16 ± 0.03	0.67 ± 0.06^*^	0.65 ± 0.03^*^	0.60 ± 0.06^*^
NORMALIZED AREA^A^	Control	1	0.61 ± 0.09	0.57 ± 0.09	0.50 ± 0.08
	Diabetic	1	1.21 ± 0.05^*^	1.18 ± 0.03^*^	1.38 ± 0.06^*^
VOLUMETRIC RR^B^	Control	2.84 ± 0.10			3.15 ± 0.05
	Diabetic	2.62 ± 0.14^*^			1.08 ± 0.05^*^

^A^ Measured by in vivo fluorescence imager

^B^ Measured by Cryo-imager

^*^ significant differences with the control (P<0.05).

### D. Histological Analysis of Wound Healing in db/db Mice

**FIGURE 7.**
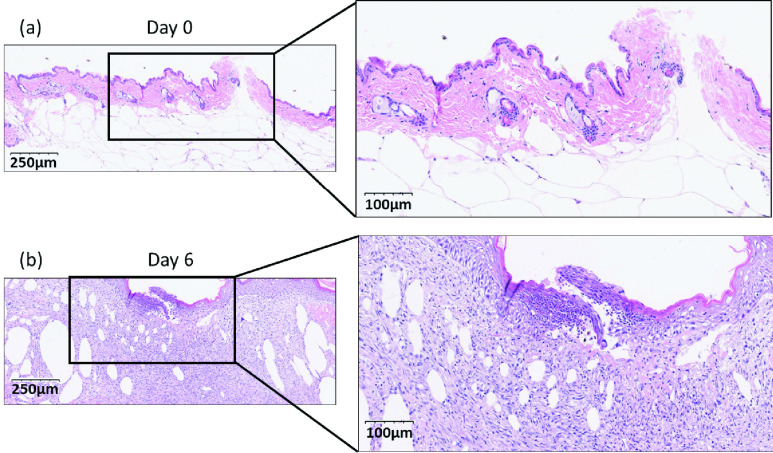
H&E stained Sections of wound tissue on diabetic mice at (a) Day0, (b) Day6 of post wounding. The comparison between day 0 and day 6 of post-wounding indicates a prolonged informatory response and disrupted wound healing.

H & E stained sections were compared between day 0 and day 6 of wound tissue on diabetic mice. On day 0, the initial wound histology revealed an intact epidermis (Fig. 7a). No inflammatory cells, except for a few resident cells, were apparent in the hypodermis. Collagen fibers were well organized. At day 6, there was evidence of re-epithelialization and early granulation tissue formation supported by new immature blood vessels (Fig. 7b). We also observed an abundance of lymphocytes and macrophages interspersed with early unorganized collagen in the hypodermis, indicative of a prolonged inflammatory response and disrupted wound healing.

## IV. Discussion

The normal wound healing process, which involves hemostasis, inflammation, tissue formation, and tissue remodeling, is significantly influenced by oxidative stress and disrupted redox signaling [9]–[11]. In the hemostatic and inflammatory phases of wound healing, immune cells generate ROS at low concentrations to protect against invading microorganisms and to upregulate the cell survival signaling pathways [37], [38]. There is a fine balance between the positive role of reactive oxygen species and their harmful effects [38]. Excessive and uncontrolled generation of ROS occurs in hyperglycemic states and contributes to dysregulated inflammatory processes known to play a central role in the pathophysiology of chronic non-healing wounds [9]–[11]. Despite a large amount of research into the pathogenesis of impaired diabetic wound healing, there is still a very limited understanding of the underlying mechanisms responsible for delayed wound healing. To the best of our knowledge, this study provides the first quantitative, longitudinal examination of mitochondrial redox state *in vivo* and at cryogenic temperature. The information that we have obtained on mitochondrial bioenergetics during diabetic wound healing confirms the importance of mitochondrial function in wound healing and provides new insights into the molecular basis of wound healing.

This study investigated the temporal role and contribution of the mitochondrial redox state in the wound healing process of diabetic and non-diabetic mice. We designed and utilized an optical metabolic imaging system to monitor the metabolic state of skin wounds *in vivo*. This imaging system is portable and noninvasive. Moreover, it images the wound dimensions, as well as. mitochondrial redox state thus enabling the quantification of the rate of wound closure and metabolism simultaneously. The redox ratio changed over time and showed a significant negative correlation (R^2^ = 0.72) with wound size (Fig. 6b), suggesting that our quantitative marker of the mitochondrial redox state is predictive of the rate of wound closure.

We use the ratio of NADH/FAD as a quantitative marker for the oxidation state of the tissue [39], [40]. The individual NADH and FAD values provide similar information on the redox state as the redox ratio, and therefore, could be used to determine tissue function. The lower NADH signal and higher FAD signal observed at day 6 of diabetic wound healing indicate that in the mitochondria, there was less NAD present in its reduced form (NADH) and high FADH_2_ present in its oxidized form (FAD). This would result in a decreased pairing of electrons with hydrogen and increased leakage of electrons in the mitochondrial ETC to available oxygen and high production of ROS.

The data obtained from our *in vivo* fluorescence imaging system was also consistent with the data obtained from our 3D optical cryo-imaging system. Both systems document a transient decrease in the redox ratio of wounds in diabetes. We propose that reduced redox ratio of the diabetic wounds, which reflects the more oxidized state of diabetic wounds, may be caused by the greater degree of oxidative stress in the diabetic wound. These findings are consistent with reports of diabetes- or hyperglycemia-induced mitochondrial dysfunction and oxidative stress in the organs and cells [20], [41]. This can be extended to mitochondrial dysfunction and high oxidative stress in chronic wounds. These factors likely contribute to the profound delay in wound healing in diabetes. The surface redox ratio and volumetric redox ratio had a significant correlation (Fig. 6b). This correlation suggests the association of mitochondrial redox state of the wound surface with the redox ratio of deeper layers in the tissue.

It is worth noting that the surface redox ratio (NADH/FAD) of the wounds in diabetic animals showed a significant drop at day 2 (Table 2), and this may be associated with the increased oxidative stress in the diabetic wounds during the earlier phases of wound healing. Studies have suggested that the redox ratio is sensitive to cellular metabolic state and vascular oxygen supply [30], [42]–[44], which is altered in diabetic animals when compared to that of the controls. Further experimentation is needed to investigate the changes in the redox state of diabetic wounds during the initial hours of post wounding.

Recently, the dynamic histological and molecular events were extensively characterized in excisional wound healing in db/db mice [36]. This study quantified the wound healing process daily from wound creation to wound closure. Consistent with our results, the authors reported significantly delayed wound closure in db/db mice manifested as impaired wound contraction and the poor quality healing. Histologically, they observed deficient re-epithelialization, irregular keratinocyte arrangement and significantly thinner granulation tissue resulting in the poor healing quality of db/db wounds. We compared the histology in db /db mice at day 0 and day 6 (Fig: 7a and 7b) to investigate the role of mitochondrial redox state, which is quantified by our optical imaging systems, in the inflammation in diabetic wounds. On day 0, hypodermis has no inflammatory cells except few resident cell populations, but on day 6, hypodermis has abundant lymphocytes and macrophages, demonstrating an increased inflammatory process. When we compared these findings with the changes in the mitochondrial redox state of the wounds in diabetic mice over time, this observation further strengthens the hypothesis that there is a correlation between an altered redox state in diabetic wounds and inflammation. Excessive presence of ROS and pro-inflammatory macrophages in the diabetic wounds are correlated with persistent NLRP3 inflammasome activity. In our future studies, we are aiming at exploring and comparing redox imaging with specific inflammatory pathways [45].

Our optical metabolic imaging systems can interrogate the differences between the mean redox ratio of the whole wound on diabetic vs. control mice. Therefore, the systems cannot specifically be used to study regional changes in the dermis, epithelial tongue, or granulation tissue. Another limitation of our work is that keratin has a similar excitation and emission spectrum to the FAD, and any differences to the keratin during wound healing can affect the FAD signal. In conclusion, optical imaging provides an optical metabolic marker, redox ratio, which is sensitive to the impaired diabetic wound healing, suggesting the diagnostic potential of optical imaging systems for clinical wound care. The system is non-invasive, non-contact, and portable to hospitals and clinics for in situ wound assessment. The group is in the process of designing a prototype that can be used in clinical wounds to pilot the diagnostic potential of this novel and emerging technology. Such an investigation would be relevant to examine the efficacy of wound healing interventions targeting ROS and mitochondrial dysfunction during diabetic wound healing [38].

## Figures and Tables

**FIGURE 8.**
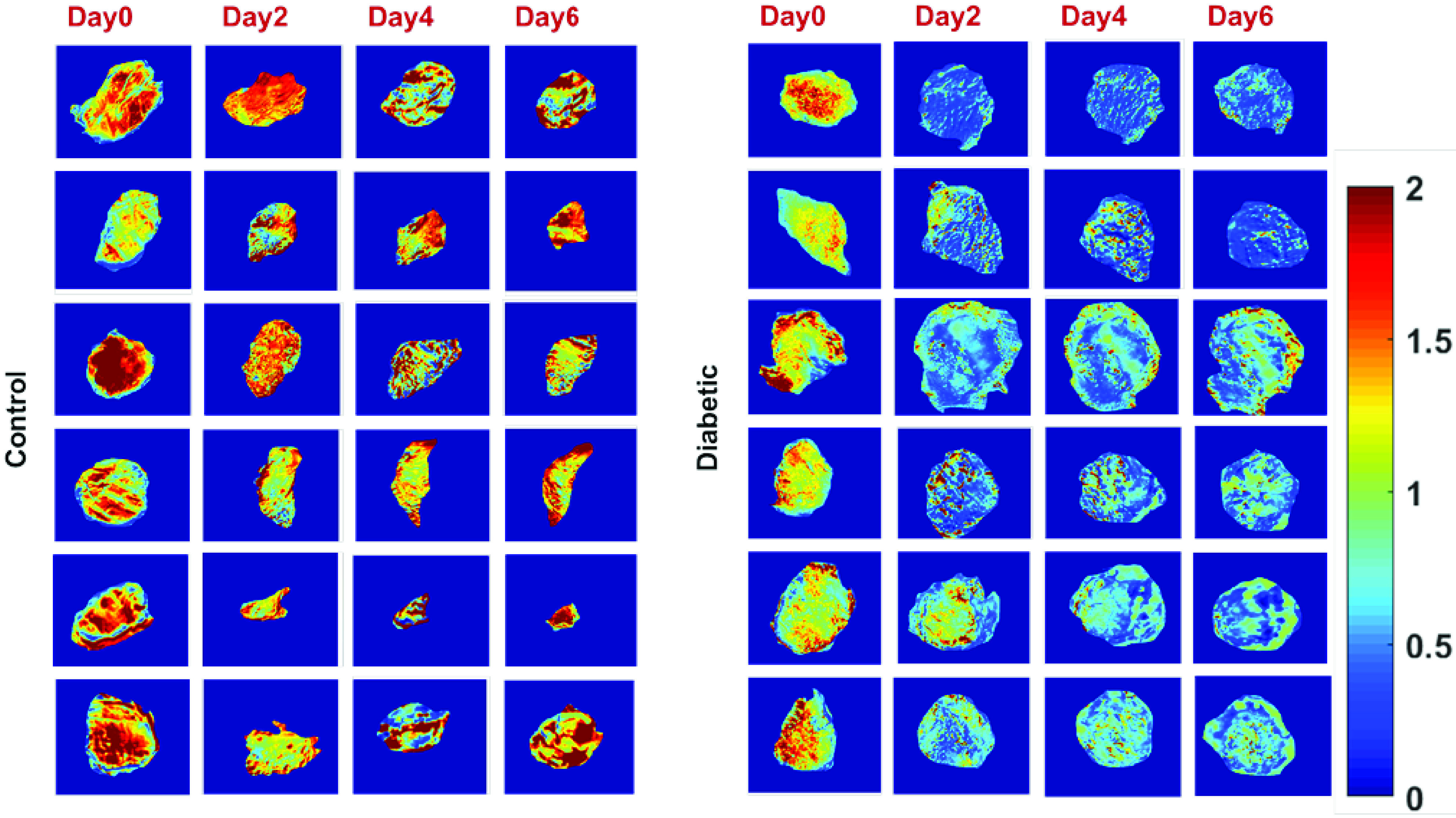
Surface redox ratio images of all wounds over days. For all mice in diabetic group, there is a consistent decrease in the redox ratio of the wounds over time. At day 6 of post punching, there is a 61% (p-value<0.001) significant difference between the mean redox ratio (surface RR) of wounds on diabetic mice in comparison with controls.

## Appendix

The redox ratio images of the wounds on individual mouse over time are shown in Fig. 8. The consistency in the redox ratio of the wounds on different mice (biological replicates) in a particular group/day signified our research findings correlating redox ratio to the wound healing status in diabetic mice.
